# A Qualitative Study on the Implementation of the Workplace TB Program in the Philippines: Challenges and Way Forward

**DOI:** 10.3390/tropicalmed8020093

**Published:** 2023-01-30

**Authors:** Evalyn A. Roxas, Vivien Fe F. Fadrilan-Camacho, Maria Margarita M. Lota, Paul Michael R. Hernandez, Adrian Paul M. Agravante, Loisse Mikaela M. Loterio, Micaela J. Arevalo, Richelle Liza F. Maglalang, Carlo R. Lumangaya, Vicente Y. Belizario

**Affiliations:** 1 Department of Medical Microbiology, College of Public Health, University of the Philippines, Manila 1000, Philippines; 2 Department of Environmental and Occupational Health, College of Public Health, University of the Philippines, Manila 1000, Philippines; 3 Department of Parasitology, College of Public Health, University of the Philippines, Manila 1000, Philippines; 4 Neglected Tropical Diseases Study Group, National Institutes of Health, University of the Philippines Manila, Manila 1000, Philippines

**Keywords:** tuberculosis, workplace, occupational health, qualitative research, Philippines

## Abstract

Tuberculosis (TB) is a chronic infectious disease that remains to be a primary health concern globally. The Philippines is among the top TB-burdened countries. Workplace TB prevention and control programs are essential to ensure the health and safety of workers and economic security. There remains a knowledge gap regarding the Philippine workplace TB prevention and control program implementation. This qualitative study involving key informant interviews reviewed the implementation of the workplace TB program in selected companies in a high TB burden region in Eastern Philippines. Results were presented under four themes in accordance with the components of the workplace TB policy: preventive strategies, medical management, data recording and reporting, and social policy. Various good practices, opportunities, and challenges in the implementation of the workplace TB program were identified. There is a need to strengthen the enforcement of policy across different components. Compliance with guidelines on preventive strategies and recording and reporting schemes needs to be intensified. Coordination across different levels and agencies may also be enhanced to allow more efficient implementation. Increased awareness of corporate decision-makers may improve company ownership of the program leading to improved implementation while increased awareness of employees on their rights and entitlements may likewise enhance compliance.

## 1. Introduction

Tuberculosis (TB), caused by *Mycobacterium tuberculosis,* is a chronic infectious disease that commonly affects the lungs but may have extrapulmonary involvement (kidney, spine, and brain) in some cases [1]. It remains to be a primary health concern globally contributing to an estimated 6.4 million TB cases in 2021. Of this, the Philippines accounts for approximately 741,000 cases (650 cases per 100,000 population) [2].

Tuberculosis, without early prevention and diagnosis, can negatively impact economic activities through workflow disruption, reduced productivity, and increased direct costs (e.g., care and treatment) and indirect costs (e.g., absenteeism and replacement and retraining of staff). TB has been estimated to cost US$ 12 billion per annum due to a decline in worker productivity [3,4]. Workplace TB prevention and control programs are therefore essential to ensure the health and safety of workers and economic security.

In the Philippines, although there is a policy on workplace TB prevention and control, there remains a knowledge gap regarding its implementation. Thus, we aimed to review the implementation of workplace TB programs in selected companies in a high TB burden region in Eastern Philippines.

### Philippine Health System and TB in the Workplace Policy

The Philippine Health System has adopted a devolved setup following the Local Government Code of 1991 [5]. The Philippine Department of Health (DOH) manages its central and regional offices while health services are delivered by local government units (LGUs) at the provincial, city, and municipal levels [6]. On the other hand, the Philippine Department of Labor and Employment (DOLE) still follows a centralized system that monitors the operation of private companies and establishments [7]. The DOLE does not have offices at the provincial, city, or municipal levels.

The National Tuberculosis Program (NTP) was established in 2003 following the Comprehensive and Unified Policy (CUP) for TB control in the Philippines [8]. The CUP places DOH at the helm of TB control activities in the Philippines with the participation of the various national government agencies including DOLE [9]. To address TB in the workplace, the DOLE has issued the “Guidelines for the Implementation of Policy and Program on TB Prevention and Control in the Workplace” which must be implemented in all establishments in the private sector [10].

The Department Order mandates including the workplace TB prevention and control program in a company’s occupational safety and health (OSH) and other related workplace programs which must be overseen by the workplace health and safety committee. The sections of the policy include: (1) preventive strategies such as having programs on TB advocacy, education, and training, giving proper information on ways of strengthening immune responses against TB infection, improving workplace conditions, and capacity building on TB awareness; (2) medical management; (3) recording, reporting, and setting up a database; (4) social policy; (5) roles and responsibilities of workers with TB or at risk for TB; (6) roles and responsibilities of employers; and (7) implementation and monitoring, with the first four being the main components of the program.

The preventive strategies component pertains to provisions on the conduct of TB advocacy, education, and training along with improving workplace conditions. The medical management section contains provisions for the adoption of directly observed therapy (DOTS) at the workplace, the establishment of compensation for TB benefits, and referral of TB cases for appropriate care through private or public health providers under the jurisdiction of DOH in compliance with the CUP. For recording and reporting, companies are mandated to include diagnosed TB cases in their reporting of illnesses and injuries in the workplace. Social policies must consider non-discrimination, work accommodation, and work restoration for TB-positive employees [10].

In addition to this, the DOH has released the 6th edition of the NTP Manual of Procedures in 2020, which covers guidelines on patient-centered TB care, screening and diagnosis, treatment, prevention, and recording and reporting of data [11].

## 2. Materials and Methods

### 2.1. Study Design and Population

This qualitative study involving key informant interviews (KIIs) was conducted in two cities and two municipalities in a high TB burden region in Eastern Philippines.

Key informants were purposively sampled to include representatives (n = 18) from relevant government agencies (i.e., DOH and DOLE) at the national and regional levels, concerned LGUs, and pilot companies involved in the implementation of the workplace TB program (Table 1). The four selected companies had at least 100 employees and a workplace TB program. Participant recruitment involved invitation letters sent via email.

In the recruitment of participants for the key informant interviews (KII), letters of invitation, together with the research proposal and ethics approval of the study, were sent to relevant national government agencies and their regional offices, concerned LGUs, and participating companies through email. Informed Consent Forms (ICFs) were sent to the identified representatives.

### 2.2. Data Collection

Pre-tested interview guides based on the provisions of the DOLE policy were utilized for the KII. For agencies and LGUs, the questions focused on their roles in the implementation of the workplace TB program. For companies, the questions included the type of industry and their policy and program on TB prevention and control.

The KIIs were held virtually via Zoom from February to March 2022. Each KII lasted for 30 to 45 min. The recorded interviews were transcribed verbatim using Microsoft Word.

### 2.3. Data Processing and Analysis

**Figure 1:**
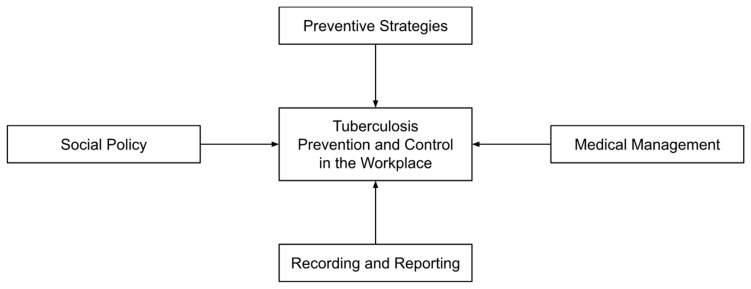
Framework for assessment of Tuberculosis Prevention and Control in the Workplace policy [10].

Deductive thematic analysis was used as described by Braun and Clarke [12]. The initial themes for the analysis were based on the components of the Philippine TB Prevention and Control in the Workplace policy [10] (Figure 1). The transcripts were coded and arranged into a framework matrix using Microsoft Excel.

### 2.4. Ethical Considerations

This study was approved by the University of the Philippines Manila Research Ethics Board (UPMREB 2021-0513-01). Prior to the interviews, informed consent was secured from the representatives. The identities of the companies and key informants remained anonymous to ensure privacy and confidentiality. Data access was restricted to the research team. No incentives or compensation were given to the participants.

## 3. Results

The data were presented under four themes in accordance with the four main components of the TB in the workplace policy: preventive strategies, medical management, data recording and reporting, and social policy, each with four subthemes (Table 2).

### 3.1. Preventive Strategies

Government agencies, LGUs, and companies collaborate with each other as well as with other groups and organizations to maximize the implementation of the TB program. Government agencies coordinate with the health offices of the LGUs on all TB activities, targets, strategies, directives, and policy updates through regular communication and meetings. They also work together in providing medications and treatment to TB patients, and in conducting training sessions and workshops for companies and local health workers. Aside from Non-Governmental Organizations, government agencies collaborate with workers and employers’ groups, and establishments for conduct of training and advocacy activities, and capacity building. Agencies also partner with academe to increase awareness on health programs. The results under this theme revealed that TB guidelines were inconsistent, and that implementation was greatly dependent on company willingness; there was poor inter-agency and inter-level coordination, and a large portion of establishments were excluded from compliance inspections.

#### 3.1.1. Inconsistencies in TB Guidelines

Challenges encountered in the enforcement include cases of inconsistencies in the treatment course, particularly in having separate guidelines for TB at the company level.

*“That’s another challenge—the private [companies] involvement in the TB [in the workplace] Program because they have other—they have separate guidelines that they follow. Although we try to meet with them so that we can include them in trainings.”*

(DOH Program Personnel, Regional Office)

#### 3.1.2. Importance of Company Support

The inclusion of the chest x-ray in the annual physical examination of employees is one of the main TB prevention and control strategies. Other measures that some companies invest in include monitoring of work environment and air ventilation, provision and use of personal protective equipment (PPE) and other technologies (such as Pi tags, enforcement of online health declarations and mandatory health cards, and promotion of healthy lifestyle and activities) which came as a result of the pandemic.

*“Due to the COVID prevention, we intensified the provision of proper ventilation of work areas, because there are some areas that we discovered are not properly ventilate[d]…I think it helped us install additional [TB] control measures for proper ventilation…We also have these Pi tags being given to our control room and power plant personnel…It is a social distancing device. If you get within one meter of each other, it will be recorded [alarm], so it is a reminder for them to keep [a] distance [of] at least one meter for COVID prevention.”*

(Health and Safety Officer, Company 4)

*“We have annual WEM, or work environment monitoring. After the results, we analyze those parameters that fail and make corrective actions…We also monitor the area for ventilation if it’s adequate or not.”*

(Health and Safety Officer, Company 1)

#### 3.1.3. Limited Conduct and Participation in Meetings and Capacity-Building Initiatives

There is reportedly limited conduct of meetings whether it be inter-agency (horizontal) or inter-level meetings (vertical) on the DOLE side of the program.

*“That’s also a slight frustration of mine. At the national level, DOH calls for regular meetings so national government agencies meet from time to time—at least at a minimum of four meetings—convene four times a year, so required once per quarter. But once you get to the regional level, it’s not like that. The enthusiasm at the national level is not mirrored at the regional level but that’s my impression. If only we could mirror the CUP (Comprehensive and Unified Policy) [on TB control] at the national level, we could translate and bring our discussions at the central office, [at the] national level, [with] national government agencies, to the grassroots, perhaps the implementation of the program from the regional office to the local government units will be more seamless.”*

(DOLE Program Personnel, National Office)

This sentiment resonated at the regional level where they rarely conduct training for the labor department.

*“So far, there isn’t [any training]. I suddenly got embarrassed. There’s none because—there isn’t anyone conducting training [for TB]. [It’s an] overall [training], not specifically for TB. So last time there was a training by [mentions office] on emerging health condition…it was like overall, not specific for TB only…In fairness, this is a good point—there’s like a gap. It’s good that DOH together with LGU has [a training] but for us [in DOLE], there’s no forever because we’re really lacking in interagency connection.”*

(DOLE Program Personnel, Regional Office)

Limited funding has been cited as a cause of the limited training being conducted.

*“Sometimes for example, there is no budget, so we conduct the training but with our resource persons or we usually do the training per province, then the resource persons for that [companies] are the provincial coordinators”*

(DOH Program Personnel, Regional Office)

On the other hand, the point was raised that company participation in agency-led seminars and workshops were also deemed as lacking.

*“Also the big ones [companies], like [company name], they can’t just attend anytime.”*

(Program Personnel, Provincial Health Office)

Other factors that were cited were high staff turnover at companies and lack of endorsement to LGU officials which hinder the invitation and attendance of these companies to program meetings and activities.

*“Yes, that’s why it [communication with companies] is a problem, their personnel gets replaced to the point that we do not know [anyone]...because we do not know to whom we will address the letter since it’s different personnel…it would be better that they inform us who the new head is so that we can [write to them], so it would not be difficult for us, we do not usually know whom we would address in the letter that’s why our [program] stopped.”*

(Program Personnel, Provincial Health Office)

On the other hand, participating companies mentioned working with their respective LGUs for information education and communication (IEC) activities which have now been discontinued. A cited reason was the onset of the COVID-19 pandemic.

*“Pre-COVID time, we partnered with the City Health Office. So in the City Health Office, they really are personnel in charge of IEC…We conducted annual lecture[s] to all workers.”*

(Health and Safety Officer, Company 4).

*“They [Rural Health Unit] used to have a team for education, everything… campaigns against TB, prevention methods… But it [RHU TB Service] has not been functioning lately because there has not been follow-up training from DOTS [center].”*

(Security, Safety, and Health Officer, Company 2)

#### 3.1.4. Missed Establishments for Inspection

According to some key informants, there was an overwhelming number of establishments to be inspected. Additionally, the workplace TB program was reportedly not widely implemented in the informal and public sector.

*“Let’s say our target is 76,000 [establishments] and let’s say we’ve surpassed that [and were] able to inspect 80,000+ establishments. But versus the universe of establishments that were given to us by the DTI (Department of Trade and Industry) of 900,000+, we’ve only roughly inspected around 10%. That’s what I was saying that most of what we haven’t been able to inspect were those that are called micro establishments because DTI said that around 94–96% are SMEs (small and mid-size enterprises). Only a few were large and medium establishments which are that we are able to cover since they have a lot of workers.”*

(DOLE Program Personnel, National Office)

Apart from these, there were reportedly even a number of establishments that were not registered under DOLE which were immediately missed by inspection.

*“Apart from these, another challenge were those that are not listed under DOLE…which is the basis of our inspection since that is the database [for inspection] by our field offices and regional offices…These micro establishments are missed not only for TB but also for other programs under the DOLE. Even until now, we are perplexed on how we will reach them. It’s very hard to find them.”*

(DOLE Program Personnel, National Office)

### 3.2. Medical Management

In the management of TB cases among employees, the companies provide medications, monetary and health service compensation, and work accommodations. The companies enroll their employees in health maintenance organizations (HMOs), Philippine Health Insurance Corporation (PhilHealth), and the Philippine Social Security System (SSS) to avail of benefits. These include reimbursement and extension of care to the dependents of the infected worker. Some companies also extend their support to nearby communities by providing medicines and implementing health surveys and outreach programs. The findings under this theme revealed that there was limited personnel for the implementation of the TB element in the workplace program, the availability of resources varied, and there were issues with high pill burden, along with service delivery challenges amidst the pandemic.

#### 3.2.1. Limited Personnel

Another challenge is the lack of health human resources, which can lead to overworked doctors and healthcare workers. Instances where manpower was not enough to focus on TB initiatives and programs, resulting in the unsustainability of projects, were recalled.

*“The thing is, the CHD level is like the overall monitoring of the program… usually the CHD NTP coordinator, they still have other programs to handle…yes it’s the personnel/people that would help or would focus on the initiatives because they could no longer focus or look into the other health programs, especially now that we have the COVID-19 pandemic. That’s why the other programs are neglected, not only with TB.”*

(DOH Program Personnel—01, National Office)

On the other hand, employers were reportedly also challenged when employees were on sick leave due to being TB-positive. Some were reportedly given as much as one month of leave. This was a burden for employers as it would negatively affect their company operations.

*“For example, he’s a skilled worker. He is really needed. So how are we going to balance? He cannot report to work first. We inform his immediate supervisor that he should not report to work… We talk to the management to always consider the employee first… Health and safety first over productivity.”*

(Health and Safety Officer, Company 4)

#### 3.2.2. Resource Availability

Despite TB being one of the better-funded programs (DOH Program Personnel—03, National Office) and the provision of funding and medical supplies by government agencies, there were a few instances when LGUs lacked medication for TB patients and their contacts.

*“The availability of the medicine, [that’s a challenge] because for instance, there’s someone diagnosed but [it falls to] the availability of free medicine. We have TB DOTS facilities wherein they cater for free. The tendency for others is that they don’t have [medicines] available.”*

(DOLE Program Personnel, Regional Office)

This resulted in out-of-pocket expenditures among patients and the reallocation of the LGUs’ budget to the procurement of TB medication. It was also mentioned that there could have been more equipment and resources that would support efficient TB diagnosis and treatment services.

*“Funding for PTB (pulmonary tuberculosis), for treatment…We are lacking in medicines… [If that happens, these will be] out of pocket.”*

(Program Nurse Coordinator, Municipality 2)

#### 3.2.3. Issues with High Pill Burden

In terms of drug adherence, issues have been raised regarding the dosage of the treatment with the multiple capsules being taken.

*“They don’t like that—15 capsules. ‘There should be 3-in-1 like that.’ So others, they’re very choosy.”*

(DOLE Program Personnel, Regional Office)

#### 3.2.4. Service Delivery Challenges in the Pandemic

Challenges due to the COVID-19 pandemic were also faced. Service delivery, advocacy, and training have been negatively affected, hindering progress toward performance targets of the TB program. During the pandemic, the provision of tablets for treatments was reportedly limited to weekly instead of every day in view of travel restrictions.

*“…the treatment partners were able to adjust. If before they gave [tablets] every day, in the pandemic they gave weekly as to limit [patients] going out [of their homes].”*

(DOLE Program Personnel, National Office)

*“Last year we were not able to participate [in TB active case finding activities], because the mayor did not permit…They [active case finding activities] can cause crowding.”*

(NTP Nurse Coordinator, Municipality 1)

### 3.3. Recording and Reporting

Monitoring of TB cases in DOTS facilities and in companies is handled by the health offices of the LGUs. Cases referred to TB DOTS facilities and local health units are reported to the DOH through an online information system. Annually, companies submit medical reports mainly to the DOLE for recording and analysis. These reports contain health-related information about the companies, which includes data on the number of employees infected with diseases such as TB. In terms of recording and reporting, there were reported limitations in programmatic coordination and data integration, in the jurisdiction of DOH on establishments, in compliance with reporting schemes, and in the completeness of the data being submitted.

#### 3.3.1. Limited Programmatic Coordination and Data Integration

As indicated by the key informants, interagency connection and proper coordination between health offices and government agencies for better implementation of the TB in the workplace program were perceived as lacking.

*“The information system, how we get the data from DOLE, from workplaces… the data for TB… which is supposed to be integrated, which has a component for integration. We also have [a] connection with SSS, GSIS. That is what is still being fixed in the information system.”*

(DOH Program Personnel—01, National Office)

This was perceived as a challenge especially for occupational health wherein the approach should reportedly be more horizontal than disease-specific.

*“Even us, we cannot figure out because the implementation of the program in DOH is very vertical. So when I say vertical, it is disease-specific. So in the infectious disease division, they have these diseases of public health significance that they focus on, like for example here we are talking about TB. And also, the strategy of DOH, it is community-based. Every service and strategy would be implemented starting at the community level, that’s why we have the rural health units, our government hospitals who provide services for these. So that is the slight gray area with us, our interface when it comes to the occupational health and TB program, because they are disease-specific while OH (occupational health) [is not]…”*

(DOH Program Personnel—02, National Office)

They also mentioned a gap in communication between workplaces, health facilities, and LGUs for monitoring TB patients.

*“Of course, DOH is advocating for coordination at all levels, so the workplace should have coordination with—they should have a partnership with a health facility, coordination with the LGU, because when it comes to the permits—health permits, you get it from the LGU, and then, with the CHD technical assistance, so there. We really need partnerships from all levels. But with DOH, in the LGU, there is no direct [connection] with them, it is with the CHD that we are connected with.”*

(DOH Program Personnel—01, National Office)

Companies coordinate with the LGUs in the referral of employees who tested positive for TB to DOTS facilities and government hospitals, treatment, and monitoring of the infected workers, contact tracing and case finding, and also in screening and diagnosis. However, there is reportedly a need for more intensified involvement from companies. Only some companies reportedly submit and share these medical records to DOH, LGUs, and TB DOTS centers.

*“What they are saying is there are no more cases there are none, so I said, why don’t we, we coordinate with these companies, because sometimes, that’s the thing, they are not… what do you call it, they are not that involved.”*

(DOH Program Personnel, Regional Office)

*“We have direct communication with the local government unit because we know that they also have TB DOTS. That would be first [we contact]. Then usually with the provincial health office since our physician is connected with them… Not much coordination with DOH…With DOLE, we submit reports like the annual medical report.”*

(Health and Safety Officer, Company 2)

There was previously an inter-agency platform for the TB in the workplace program but it was interrupted by the COVID-19 pandemic.

*“Here in DOH, they are part of the RCC—Regional Coordinating Committee along with DOLE. They have a meeting every quarter. That’s where they have coordination for the program and between agencies…[However,] the last meeting of the RCC was still in February 2020. Maybe they need to re-activate it and push the TB in the workplace program.”*

(DOH Program Personnel—03, National Office)

#### 3.3.2. Limited Jurisdiction by DOH on Establishments

There was reportedly limited jurisdiction of the DOH in the workplaces which was perceived to be a challenge towards compliance and data reporting.

*“The information gathered from private entities were limited. If DOLE were to spearhead a program with strict monitoring on medical checkups of companies for employees, there is limited power of DOH over those facilities. Maybe [it would be good] if DOH would be given linkage or authority by DOLE.”*

(DOH Program Personnel—03, National Office)

#### 3.3.3. Limited Compliance

Noncompliance with the guidelines and procedures was also reported, particularly in the process of referral of TB cases from companies to the LGUs. It was mentioned that there are instances that official forms were not being used despite being endorsed by the rural health unit (RHU).

*“For instance, when there are findings in the [chest] x-ray, they [companies] refer [to physicians] but they do not have a referral form. Official referral forms are distributed to them, but they remain unused.”*

(Program Nurse Coordinator, Municipality 2)

#### 3.3.4. Incomplete Data Reported

There is reportedly incomplete data being submitted by reporting units. This reportedly makes it difficult to track patients and trace the origin of infection. Moreover, current incidence rates could reportedly be underestimated.

*“It’s either the name of the facility, or the source of the TB case. But the thing is, there are facilities that don’t include the name of the facility, so you don’t really get the complete picture of it is from the workplace or not.”*

(DOH Program Personnel—01, National Office)

*“It’s not [accurate] anymore…[It] could be underreporting.”*

(DOH Program Personnel—01, National Office)

For micro-, small-, and medium-sized enterprises (MSMEs), limitations in the DOLE database were reported in the case of unregistered MSMEs. These MSMEs reportedly have little resources to implement the TB in the workplace program.

*“The private sector is a different thing, because while this is institutionalized in the private sector, the problem is with micro enterprises, they are required to have OSH established in their workplace or establishment. Although it will entail resources that the medium, small, and micro enterprises don’t have.”*

(DOH Program Personnel—02, National Office)

The public sector was reportedly another gap in terms of implementation. Institutionalizing the TB in the workplace program in the public sector has reportedly yet to be established.

*“The challenge with that I think is…the public sector since it’s not yet established and with the medium, small and micro enterprises, how or where will they go for these services…So with the surveillance part, I don’t know how you would implement it for those that don’t have the established system, for example in the private medium, small or micro, [and] in the informal sector...”*

(DOH Program Personnel—02, National Office)

A key informant also noted that there should be proper enforcement of policies including penalties for noncompliance.

*“I’m also not sure if they are also issuing notices of violation, if they don’t submit will they issue something…Our policies should have fangs, because so what if we will follow or not. If you would say that it’s for your own good, it’s for your employees’ health, welfare, and things like that, but those are just, so to speak, additional burden, except maybe for the big companies, it’s quite manageable, but that’s the thing, who will monitor…There are already templated policies…there is just a need for it to have claws, for someone to enforce them.”*

(DOH Program Personnel, Regional Office)

A key informant from the labor department also noted that there is a need to update the reporting system to allow disaggregation of data. This will reportedly allow them to identify which agencies should be given more attention and assistance.

*“Age is also not there [in the reporting system]. We have no means to identify and that is still a challenge for us. I keep telling DOH whenever they say that there is missing [data] from us that if they improve their reporting system—put if the patient is working or not, what industry they’re in—then we could identify which industries we need to give attention.”*

(DOLE Program Personnel, National Office)

### 3.4. Social Policy

As part of their policy, the DOLE ensures the safety, health, and protection of employees, and respects the rights of patients with regard to psychosocial needs. Companies, in coordination with stakeholders, also see to it that the medical condition of their employees remains private and confidential. Under this theme, the subthemes that emerged highlighted the importance of social policy provisions and consultation with experts along with challenges on stigma against TB-positive individuals and limited knowledge on TB benefits.

#### 3.4.1. Importance of Social Policy Provisions

Respondents recognize that there are social concerns that are related to availing of TB services for the working population. For instance, employees who were being treated for TB reportedly had challenges at work with visiting treatment facilities during workdays. A cited good practice was having provisions for adjusting work hours for employees.

*“Of course, it’s not only a health issue. It also includes rights issues—that you won’t be suddenly terminated from work just because you have TB, which is curable—so it also includes issues against discrimination and of course work accommodation for days that you will need to submit sputum, do follow-up check-ups with a DOTS physician. Flexibility will be given by the workplace either flexi-time or earlier work shifts so that the patient could complete the eight work hours and go to the DOTS facility or it could also be in between [work hours].”*

(DOLE Program Personnel, Regional Office)

Companies also recognized this and provided the necessary support for their TB-positive workers, especially during treatment and recovery of those workers.

*“For example, if the employee is in a graveyard shift, we have to take care of that and take into consideration all the environmental factors that will speed up his healing. We assign him to a work shift that is most convenient to him while recovering.”*

(Health and Safety Officer, Company 1)

#### 3.4.2. Stigma against TB-Positive Individuals

With reported instances of stigma towards TB, attention and perceived importance of TB response in barangays (villages) were noted as lacking.

*“The stigma of having TB is still there. When they get infected with TB, they are ashamed to let their community know that they have the TB disease.”*

(City health program personnel, LGU 4)

Companies also encountered challenges in fighting the stigma against TB. They addressed this by educating their workers through IEC materials and town hall meetings. Some developed guidelines on TB which included non-discrimination of TB-positive workers.

*“Actually we have infectious disease guidelines in our head office. There’s the “no discrimination” policy among HIV and TB. So we have these guidelines installed... As long as they are properly treated and have obtained clearance, they can go back to work.”*

(Health and Safety Officer, Company 4)

#### 3.4.3. Limited Knowledge on TB Benefits

Another challenge identified by the stakeholders is the gap in knowledge of workers on TB and other OHS programs, particularly on the benefits of TB-positive employees.

*“There are instances that they [employees] are not fully aware that once you’re a TB patient, you can file it under SSS [Social Security System]. So, for instance, they don’t—all they focus on is the negative—the negative aspect, ‘Oh no, another expense, oh no, I cannot work,’ but the positive [aspect] is that medications are free, then they also have compensation from SSS. So yes, it’s more on awareness, proper… information dissemination is what’s needed.”*

(DOLE Program Personnel, Regional Office)

In companies, there were also instances where employees insisted on returning to work, despite being reported as still unfit to work. This remains a challenge not only to companies but also to the treatment partners, as TB treatment policy requires at least 2 weeks of treatment and certification before an employee can resume work [11].

#### 3.4.4. Consultation with Experts

There was a reported consultation with technical experts on the IEC materials in recognition of their expertise on behavior change.

*“We also have templates on posters and IEC materials but of course we work with other partners on these types of things since this is their specialty—how to work within sociocultural norms, behavioral change communication. They’re more experienced in that.”*

(DOLE Program Personnel, National Office)

Some companies maintained close relationships with agencies to assist them in conducting training and seminars on health including infectious diseases such as TB.

*“We also invite somebody from external agencies to lecture in the seminar. We invite everybody… We also invite the LGUs to lecture. For the contractors, they also have their separate lecture if they want. But we invite everyone for the lectures… Sometimes the DOH invites us whenever they have seminars”*

(Health and Safety Officer, Company 4)

## 4. Discussion

The findings of the study revealed various opportunities and challenges in the implementation of the TB in the workplace program. Among the opportunities were the importance of company support, social policy provisions, and consultation with experts. On the other hand, challenges that were identified were inconsistencies in TB guidelines along with limitations in compliance, personnel, data availability and completeness, awareness, and collaboration, among others.

Key informants reported that there were inconsistencies between company guidelines and national standards. This was similar to the findings of a qualitative study in South Africa where program implementors at the primary healthcare level encountered guidelines conflicting with national standards. In addition, South African program implementors reported a lack of training on the guidelines resulting in their limited understanding of its provisions [13]. Respondents in this study also cited the limited training for the TB in the workplace program which resulted in implementation challenges. In 2015, only one of five establishments provided trainings/seminars on TB prevention and control, ranking 8th among the top OHS trainings/seminars availed by employees [14]. More recent data revealed that TB prevention and control seminars for employees were still not among the five most conducted OHS trainings/seminars by companies [15]. Other studies also reported similar challenges with limited training for health workers [16] and long intervals between refreshers [13].

There was reportedly limited coordination on the implementation of the TB in the workplace program. Although meetings were reportedly regular at the national level, meetings were reportedly seldom done at the lower levels. A local qualitative study on the implementation of another inter-agency program (antimicrobial resistance program) also reported a similar finding on the limited coordination across different levels of implementation and among relevant national agencies [17]. The former was linked to devolution which fragmented the health system into thousands of units that lacked coordination and varied greatly in resources for health [18].

Moreover, there were limited and overworked health human resources along with an inadequate supply of drugs for TB in some facilities which challenged service delivery. These findings were supported by a local survey where insufficient human resources and drug availability were among the cited health-system-related barriers to TB control. The barriers observed in the survey may reportedly have been due to varying prioritization of health across LGUs [19]. Another local study on TB treatment in drug abuse treatment and rehabilitation centers (DATRC) added that TB drug procurement differs per facility which results in varying drug availability [20]. The delivery of TB services was also heavily affected by the COVID-19 pandemic which was observed at national [21] and global levels [22]. In 2020, the WHO estimated a 37% shortfall in the TB case notification rate in the Philippines [22]. Following this, an 18% increase in TB treatment was seen in 2021 compared to 2020 as the DOH seeks to catch up with the gains of the NTP [21].

According to a key informant, there were accounts of missing important patient data such as age and sex. This was also observed in private practice where a much earlier study among private practitioners revealed that standardized recording of TB patients and obligatory reporting of new TB cases were hardly followed [23]. When available, data on occupational diseases including TB was rarely disaggregated by size of company [24]. Moreover, there is reportedly limited compliance in the reporting scheme which was also observed in the qualitative study on DATRC where the majority of the included facilities had no recording or reporting system for TB patients [20]. According to Hernandez and colleagues, there is a widely-recognized challenge of record-keeping for OHS in Philippine industries. The reported limited integration of information systems was also described in a local study that reviewed records on OHS [25]. This may be contributing to the challenges in communication across workplaces, health facilities, and relevant national agencies.

The findings of the study have highlighted how social policy provisions contributed to patient compliance. A modelling analysis estimated that there would be a reduction of 76.1% in the global TB burden, if social protection coverage was expanded [26]. A qualitative study in South Africa revealed that compensation for work-acquired TB infection was a motivator for TB screening at the primary healthcare level [13]. However, some challenges were reported regarding the limited knowledge of employees on their rights and entitlements. The study’s findings also highlight the importance of technical expertise in developing effective health education materials.

There was an evident challenge in monitoring companies, especially MSMEs. A review conducted by the International Labour Organization revealed that policies and OHS research left out a large portion of MSMEs due to their heterogeneous nature, wide dispersion, lack of cohesive representation, and short life spans [24]. Another study also cited the limited capacity of ministries of labour to inspect sites outside of cities and major towns [27]. This challenged the coverage of MSMEs in inspections and interventions resulting in the limited availability of statistics among MSMEs [24].

Considering the reported inconsistencies in the implementation of TB in the workplace guidelines, the labor and health sectors must intensify the enforcement among companies to comply with the DOLE policy. As several key informants noted some missed establishments for inspection, the enforcement of the DOLE OHS Standards must also be intensified. Efforts may be made in including a Certification of Compliance to OHS standards as part of the requirements of Business Permit Renewals to aid in monitoring and shared responsibility between the business and the government. On the other hand, there is a monitoring gap for those outside the database which puts emphasis on intensifying policing of non-registered establishments by the labor department. With regard to data recording and reporting issues, there is a need for stricter enforcement of data recording and reporting schemes to ensure appropriate referral and management of suspected TB cases. The possibility of the labor department deputizing LGUs, particularly sanitary inspectors along with other local health program implementors, to inspect establishments for issuance of licenses to operate may be explored as is done by the Food and Drug Administration for MSMEs with low-risk products [28]. There is also a need to improve the completeness of data being reported. There is a need to include these variables in recording schemes to enable disaggregation of data. Data disaggregation allows the characterization of factors contributing to transmission and identification of vulnerable populations at most need of attention [29]. Data must also be made available to all concerned agencies to guide their implementation. The use of modern digital health tools and recording systems may help to expedite reporting and inform service delivery [30].

Seminars and orientations on employee TB benefits may be required to boost employee awareness and treatment compliance. Counseling may also be offered by workplaces which may serve as an avenue to help employees navigate their social protection system [31]. Stigma must also be addressed for communicable diseases such as TB and COVID-19. Stigma and fear may cause people to mask health conditions to avoid discrimination, delay health-seeking, and reject healthy behavior [32]. Health education and responsible social media communication are necessary to address social stigma and discriminatory behavior that negatively impact communicable disease prevention and control [22]. Company ownership of TB workplace programs must be increased and sustained. Apart from training health and safety officials, the labor and health departments may conduct health education of key corporate decision-makers on how health targets align with corporate goals. When a business invests in the health of its employees, it boosts worker capacity and productivity in the short run and decreases healthcare costs in the long run [33]. This creates a cycle of health and productivity.

Given the devolved health system in the Philippines, it is essential to improve coordination through the conduct of meetings and capacity-building activities across all levels especially as you reach the grassroots where implementation occurs. Meetings should not only be vertical in nature but should be horizontal as well, engaging both the health and labor departments [30]. These inter-agency meetings will help foster collaboration and align activities toward achieving targets. Platforms for interagency collaboration may need to be restored or established to allow coordinated action by the labor department, the health department, and the LGUs [30].

Finally, ensuring adequate resources and capacity at different levels of implementation is essential. The similarities between TB and COVID-19 diagnosis and preventive strategies have led to a growing recognition that integration of such interventions may be the way forward [34,35]. The financial and workforce resources for these two programs may be integrated for more efficient allocation and implementation [36]. A similar rigor in the enforcement and assessment of compliance to COVID-19 guidelines and protocols in the context of business operations may be adopted for the TB in the workplace program.

To the knowledge of the authors, this is the first qualitative study on the implementation of the TB in the workplace program in the Philippines across levels and agencies. This study also provides additional insight to the challenges and opportunities in the implementation of the TB in the workplace program during the COVID-19 pandemic. However, some limitations in the study include possible selection bias among the key informants for the interviews as the participants were identified by the agency, LGU, or company which they were part of, and not by the researchers, which could have affected the representativeness of the sample. Social desirability bias may also have been present during the interviews for there could have been a tendency for respondents to give answers that are more socially acceptable. The information gathered was self-reported, and thus may not have been fully accurate. Overreporting of practices that are adherent to the principles of the workplace TB program may have also occurred especially given that the study is concerned with the implementation of policy and program of TB prevention and control. These problems were minimized by triangulation, probing on responses, and confirming information with other key informants.

## 5. Conclusions

Various good practices, opportunities, and challenges in the implementation of the TB in the workplace program were identified. There is a need to strengthen the enforcement of policy across different program components. Compliance with guidelines on preventive strategies and recording and reporting schemes needs to be intensified. Coordination across different levels and agencies may also be enhanced to allow more efficient implementation. Increased awareness of company decision-makers may improve ownership of the program leading to improved implementation while increased awareness of employees on their rights and entitlements may likewise enhance compliance.

## Figures and Tables

**Table 1:** Key informants by sector and level, February–March 2022.

	Health	Labor *	Other
National	3	1	n/a
Regional	1	1	n/a
Provincial	2	n/a	n/a
City/Municipality	5	n/a	n/a
Company	n/a	n/a	5

* There are no provincial and city/municipal labor department offices.

**Table 2:** Summary of themes and subthemes.

Theme	Subthemes
Preventive Strategies	○ Inconsistencies in TB guidelines ○ Importance of company support ○ Limited conduct and participation in meetings and capacity-building initiatives ○ Missed establishments for inspection
Medical Management	○ Limited personnel ○ Resource availability ○ Issues with high pill burden ○ Service delivery challenges in the pandemic
Recording and Reporting	○ Limited programmatic coordination and data integration ○ Limited jurisdiction by DOH on establishments ○ Limited compliance ○ Incomplete data reported
Social Policy	○ Importance of social policy provisions ○ Stigma against TB-positive individuals ○ Limited knowledge on TB benefits ○ Consultation with experts

## Data Availability

Not applicable.
